# The Bioactivity and Physicochemical Properties of Emulsions Based on Tamanu, Moringa, and Inca Inchi Oils

**DOI:** 10.3390/foods13010062

**Published:** 2023-12-22

**Authors:** Aleksandra Makiej, Zofia Hochór, Wojciech Smułek, Ewa Kaczorek

**Affiliations:** Institute of Chemical Technology and Engineering, Poznan University of Technology, Berdychowo 4, 60-695 Poznan, Poland; aleksandra.makiej@doctorate.put.poznan.pl (A.M.); ewa.kaczorek@put.poznan.pl (E.K.)

**Keywords:** plant-derived oils, tamanu oil, inca inchi oil, moringa oil, antimicrobial properties, emulsion stability, natural stabilizers

## Abstract

With increasing bacterial resistance to antibiotics, novel strategies for protection against microbial infections are crucial. Emulsions enhance the solubility of natural antibacterial oils and their uptake, making them promising drug delivery systems. However, it is important to find the right emulsifier to ensure that the oil has the right dispersion and does not adversely affect its antibacterial properties. Hence, this study investigated emulsions created from three vegetable oils: moringa oil from *Moringa oleifera* seeds, inca inchi oil from *Plukenetia volubilis* seeds, and tamanu oil from the *Calophyllum inophyllum* fruit. Emulsions were formed using two natural emulsifiers, lecithin and casein, at concentrations of 2.5%, 5%, and 10% (*w*/*w*). The study assessed the oil and emulsions’ characteristics, including the zeta potential, creaming index, and particle size distribution. The antimicrobial properties of these oils and the most stable emulsions were examined. Gas chromatography was used to analyze the oil compositions. The potential antimicrobial properties of emulsions formulated with natural oils was proved. Particularly noteworthy were emulsions containing a 2.5% inca inchi or tamanu oil, stabilized with casein. The particle size ranged between 100 nm and 900 nm with the average size 300 nm. These emulsions also showed antibacterial activity against selected strains, and the strongest effect was observed for the system with inca inchi oil, which reduced *S. epidermidis* bacterial activity by more than 60%. Therefore, it can be expected that the completed research will allow the development of antibacterial systems based on inca inchi or tamanu oils for use in the food industry.

## 1. Introduction

In last decade, industry and science researchers have paid a lot of attention to new vegetable oils as a dispersed phase in emulsions. Some interesting examples of vegetable oils are moringa oil, tamanu oil, and inca inchi oil. These are gaining popularity in the food industries; therefore, they were taken into further examination as a subject of this research to obtain stable emulsions with antimicrobial properties [1,2].

The first of the oils listed above, moringa oil, is extracted from a tropical tree—moringa oleifera (*Moringa oleifera*)—which grows primarily in the Middle East as well as in Asian and African countries. Of particular interest is the oil extracted from the seeds [3]. The content of saturated fatty acids totals 18.8% (palm acid, stearic acid, peanut acid, and behenic acid). Unsaturated fatty acids account for 81.2% of the content, and oleic acid dominates, accounting for as much as 70.2% of the composition. *M. oleifera* seeds are also a rich source of bioactive compounds such as alkaloids, flavonoids, and phenolic acids, which are attributed to pharmacological properties for ailments like stomach or joint pain, as well as digestive support [4]. The seed extract has also been shown to exhibit antibacterial activity against numerous species of bacteria and fungi. The antimicrobial activity is caused by the presence of a short cationic protein responsible for damaging bacterial cells [3].

In turn, tamanu oil is obtained from the fruit of the *Calophyllum inophyllum* and consists mainly of saturated fatty acids (41–52%), and stearic acid dominates (25–35%). Unsaturated fatty acids account for about 18–22% of the fat fraction composition. Tamanu oil is characterized by the presence of a resinous part, which is unparalleled in any other oil of plant origin. It accounts for about 10–20% of the oil and is soluble in ethanol. Its composition consists mainly of neoflavonoids and pyranocoumarin derivatives. It has found applications in rheumatism and sciatica because of its pain-relieving properties and in wound healing because of its analgesic properties [5,6]. Tamanu oil also exhibits antibacterial and antifungal properties. Particularly high antibacterial activity has been noted for strains of bacteria associated with skin diseases such as *Staphylococcus aureus*, *Bacillus cereus*, *Staphylococcus epidermidis*, *Staphylococcus haemolyticus*, and *Corynebacterium minutissmimum*. Its antimicrobial activity has been attributed to some of the neoflavonoids present in the oil, particularly calophyllide and inophyllum C [6,7].

Moreover, inca inchi oil, also known as sacha inchi oil, is extracted from the seeds of the Peruvian *Plukenetia volubilis* plant. Inca inchi oil is characterized by a high content of unsaturated fatty acids, phenolic compounds, and vitamin E. The composition of the fat fraction is dominated by alpha-linolenic (40.47%) and linoleic (37.33%) acids. Saturated acids account for 8.86% of the fraction, unsaturated acids account for 13.08% of the fraction, while the content of polyunsaturated fatty acids accounts for as much as 77.80% of the oil’s composition [8]. Little information is currently available on the antibacterial properties of inca inchi oil. The effect of *Plukenetia volubilis* against three strains of bacteria, *Staphylococcus aureus*, *Staphylococcus epidermidis*, and *Propionibacterium acnes*, has been studied, where sacha inchi oil showed no antibacterial properties. In contrast, studies of the effect of sacha inchi oil on reducing the adhesion of *Staphylococcus aureus* bacteria to human skin and keratinocytes were favorable [9,10,11].

Emulsion systems can be a favorable solution for the delivery of numerous substances exhibiting poor solubility in water, additionally providing better absorption. It can be assumed that further research on emulsions will contribute to the introduction of new solutions for various food applications [12]. To date, only a few papers have been devoted to the production and characterization of emulsions based on the above-mentioned oils. Pham and Nguyen [13] described the preparation of tamanu oil nanoemulsions using the relatively toxic synthetic surfactant Tween 80. The same surfactant was used by Musa et al. [14] to emulsify moringa oil. In contrast, Urbánková et al. [15] used a natural casein-based emulsifier to emulsify with tamanu oil, but this is the only paper describing the properties of such a system, in addition to not addressing the rheological properties of the emulsion. In the case of inca inchi oil, the only paper on its emulsion with natural stabilizers (maltodextrin and a modified starch) is that published by González-Cardozo et al. [16]. This scarcity of work demonstrates the need for more research on emulsions of the aforementioned oils stabilized with natural emulsifiers, which would allow their use in the food industry. Hence, the aim of this study was to investigate the antimicrobial and physical properties of different emulsions of inca inchi, tamanu, and moringa oils. To that purpose, the toxicity of the oils against microorganisms was tested, both for pure oils and oils in emulsion systems, as well. The stability of the emulsions (their creaming index) and their rheological properties were also determined.

## 2. Materials and Methods

### 2.1. Chemicals

Vegetable oils such as *Plukenetia volubilis* (inca inchi) seed oil and *Calophyllum inophyllum* (tamanu) seed oil were purchased from NACOMI GROUP Ltd. (Wilkowice, Poland), whereas the *Moringa oleifera* seed oil was purchased from Nitai Ltd. (Wroclaw, Poland). The purity and composition of all oils were investigated using gas chromatography (see Supplementary Materials). The casein and lecithin were ordered from Merck and Warsaw, Poland, respectively. For all samples containing water, ultrapure water (18 MΩ*cm, 71.98 ± 0.01 mN/m) was applied. The AlamarBlue™ cell viability reagent was delivered from Life Technologies Polska Ltd. The microbial media like the nutrient agar, yeast extract peptone dextrose agar, and the nutrient broth were purchased from BTL Ltd. (Łódź, Poland). All bacterial strains, as well as a fungal strain that was used in experiments, were collected from legitimate sources like the Polish Collection of Microorganisms (PCM) or the American Type Culture Collection (ATCC).

### 2.2. Kirby-Bauer Disk Diffusion Susceptibility Test

The toxicity effects of the chosen three oils on six bacterial strains (*Escherichia coli*, *Pseudomonas aeruginosa*, *Pseudomonas fluorescens*, *Staphylococcus epidermidis*, *Staphylococcus aureus*, and *Bacillus cereus*) as well as on one fungus model of cells (*Candida albicans*) were evaluated by performing antibiograms. These microorganisms were chosen because of their prevalence in the environment as a cause of infection and the fact that they are common reference strains for various biocidal compound tests.

The antibiograms were analyzed according to the EUCAST recommendations for the bacterial susceptibility disk diffusion method [17] and were done on Petri dishes with nutrient agar for bacterial strains, and yeast extract peptone dextrose agar (YEPD; 2% BactoTM peptone, 2% dextrose, 1% yeast extract, 2% agar) for the fungal strain. For this purpose, bacterial suspensions with an optical density of 0.5 on the McFarland scale were obtained by mixing mineral medium (with a composition of Na_2_HPO_4_ × 12 H_2_O: 14 g/L, KH_2_PO_4_: 2.8 g/L, NaCl: 0.5 g/L, NH_4_Cl: 1.0 g/L) together with a colony of the selected microbial strain collected using a sterile cotton swab. Optical density was measured using a DEN-1 densitometer (Grant Inc., Beaver Falls, PA, USA).

Using sterile cotton swabs, the appropriate microbes’ suspensions were taken and cultures were spread evenly on the whole area of sterile Petri dishes filled with properly selected agar (described above). Sterile paper disks were then soaked in 30 μL of the selected oils and applied with sterile tweezers on the previously cultured dishes (at an interval of no more than 15 min after culturing the colonies).

The plates with bacteria were then incubated for 24 h at 37 °C, while those with the fungal strain were incubated for 48 h at 30 °C. After this time, the inhibition zones formed around the discs were measured. The experiment was performed in triplicate.

### 2.3. Emulsion Preparation

In the next stage, a series of oil-in-water emulsions were prepared. Therefore, vegetable oils were added separately to test tubes in a rich 2.5%, 5%, and 10% (*v*/*v*). Subsequently, lecithin was added to one of the tubes as an emulsifier and casein to the other. In the case of the lecithin emulsions, the first step was to dissolve 1 g of lecithin in 1 mL of water until a homogeneous, thick mass was obtained. Then 0.1 mL of it was taken and placed in a test tube. On the other hand, in the cases of the casein emulsions, the first 2 g of the casein was dissolved in 100 mL of water, and later a small amount of 10% NaOH was added to the solution to obtain a pH between 6 and 7 (checked with litmus paper). The prepared solution was stirred with a magnetic stirrer for 24 h, after which 0.1 mL of casein solution was taken and placed in a test tube. This pH adjustment is in line with the method described by McSweeney & O’Mahony [18] and Yao et al. [19], where water-soluble caseinate salts were prepared by treating isoelectric casein with alkali, such as NaOH, at a pH of 6.7. Then, depending on the final concentration, appropriate amounts of oil and water were added to the tubes with the lecithin and casein, respectively.

The final sample volume was 20 mL and each concentration was prepared twice. All the samples prepared in the abovementioned way were shaken for 5 min on an IKA Yellow Line TTS 2 laboratory shaker at 2500 rpm. After shaking, the test tubes were placed in a Bandelin Sonopuls HD 3100 ultrasonic homogenizer for 5 min (working parameters: 51 W with 5 s of puls time).

### 2.4. Creaming Index

In the following step, an analysis of the creaming index of the obtained emulsions was evaluated. Hence, a series of images were taken at the following time intervals: immediately after sample preparation, after 24 h, after one week, after two weeks, and after four weeks. The emulsions were stored at room temperature throughout the analysis. The ratio of the height of the oil layer, which was formed as the emulsion destabilization proceeded, to the height of the entire emulsion was then determined from the photographs. The creaming index (CI) was calculated according to the Formula (1):

(1) $${CI}_{x}=\frac{H_{l}}{H}\cdot 100\%$$

where: *x*—time interval (number of days after which the photograph was taken), *H_l_*—the height of the upper layer, *H*—total height of the system.

### 2.5. Zeta Potential and Particle Size Distribution

Subsequently, for the obtained emulsions, the analysis of the zeta potential and particle size distribution of the emulsions was assessed using a Zetasizer Nano ZS Instrument analyzer (Malvern Instruments Ltd., Malvern, UK). The particle size distribution measurement was completed in triplicate, while the zeta potential measurement was repeated five times.

### 2.6. Rheological Measurements

In addition, rheological tests of the obtained emulsions were carried out according to the methodology validated and described by Różańska et al. [20]. Briefly, the emulsion samples were placed in a water bath for a period of 60 min at 25 ± 0.5 °C. Rheological assessments under shear flow conditions were conducted employing a Physica MCR302 rheometer manufactured by Anton Paar (Austria) with a thermostatic unit (providing the heated fluid for the heating jacket). Viscosity profiles were ascertained utilizing coaxial cylinders CC27/T200/AL (in accordance with DIN EN ISO 3219 [21]), maintaining a gap distance of 1.0847 mm. Flow curves were determined at a controlled shear rate, which varied from 1.56 s^−1^ to 600 s^−1^. Shear stress registrations were made 60 s after the set shear rate was reached.

### 2.7. Biological Toxicity

The collected biological material was placed into separate tubes from previously inoculated colonies of the bacterial strains (*Escherichia coli*, *Pseudomonas fluorescens*, *Pseudomonas aeruginosa*, *Staphylococcus epidermidis*, *Staphylococcus aureus*, and *Bacillus cereus*) and the fungus (*Candida albicans*). The tubes were then flooded with nutrient broth and placed in a heated room (at 28 °C) for 24 h. The tubes with fungus were then flooded with yeast extract peptone dextrose (YEPD) liquid medium (2% BactoTM peptone, 2% dextrose, 1% yeast extract) and placed in a heated room (at 28 °C) for 48 h. After incubation under the specified conditions, a series of dilutions were made by flooding the tubes with a mineral medium (consisting of Na_2_HPO_4_ × 12 H_2_O: 14 g/L, KH_2_PO_4_: 2.8 g/L, NaCl: 0.5 g/L, NH_4_Cl: 1.0 g/L). The tubes prepared in this way were placed in a Centrifuge 5910 R, from Eppendorf (Hamburg, Germany), for 10 min. The centrifuge was operated with a relative centrifugal force (RCF) of 4500. After the samples were removed from the centrifuge, the procedure was repeated (i.e., the biological waste with the mineral medium was poured off, and the tubes were flooded with a fresh dose of mineral medium and placed back in the centrifuge for 10 min).

Sterile 96-well plates were used for the experiments. Test wells contained 160 µL of the emulsion or mineral medium and 40 µL of the microbial suspension, respectively, or 200 µL of pure oil in the case of the negative control. After preparation, the plates were incubated in the heated room (at 28 °C) for 24 h. For the biological tests, only emulsions with an oil concentration of 2.5% (*v*/*v*) were used since they showed the best stability in visual evaluation. The AlamarBlue (AB) indicator, which works on the oxidation-reduction principle, was then applied to assess the viability of cells. Thus, 20 µL of AB was added to each test well. The growth of living prokaryotic or eukaryotic cells causes resazurin to be reduced to its resorufin form, resulting in a visible color change from blue to pink.

Followingly, the absorbance of the samples was measured using a Multiskan microplate spectrophotometer from Thermo Fischer Scientific (Dreieich, Germany) for wavelengths of 570 nm. Based on the results obtained, the metabolic activity of each strain was calculated.

### 2.8. Statistical Analysis

Unless otherwise stated, the measurement series (made in triplicate) values and their related standard deviations were calculated using an average of three measurements. An ANOVA (one-way analysis of variance) was used to establish the statistical significance of differences between the mean values, with Tukey’s range test being used as a post hoc analysis. Differences were deemed statistically significant at *p* < 0.05. A GraphPadPrism was used to make the computations (GraphPad Prism version 8.4.3 for Windows, GraphPad Software, La Jolla, CA, USA, www.graphpad.com, accessed on 20 December 2023).

## 3. Results

### 3.1. Oils’ Antimicrobial Activity

**Figure 1:**
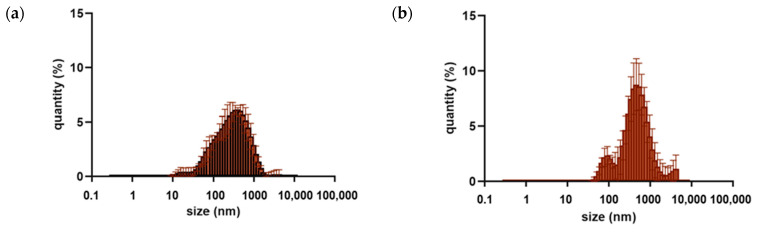

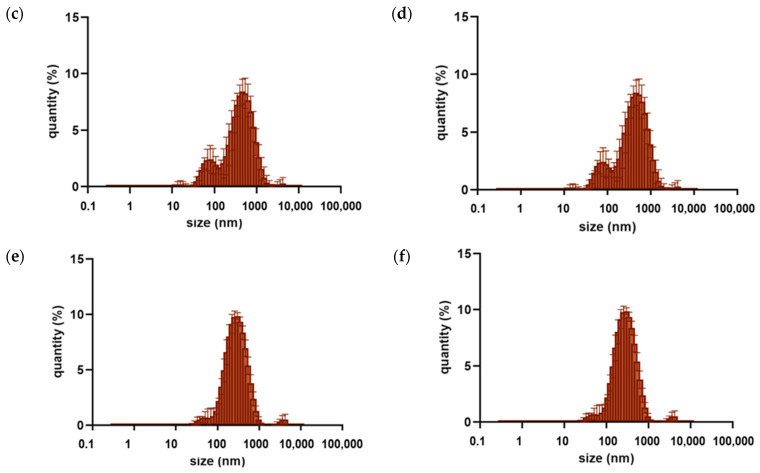
Particle size distribution for o/w emulsions with 10% oil emulsions with inca inchi oil (**a**,**b**), moringa oil (**c**,**d**), tamanu oil (**e**,**f**), each stabilized with lecithin (**a**,**c**,**e**) or casein (**b**,**d**,**f**).

Zones of growth inhibition were measured using a ruler with an accuracy of 0.5 mm, at a distance of 30 cm from the eyes, under reflected light at an angle of 45°. Photographs of selected Petri dishes are shown below in Figure 1. All trials were done in triplicate. The measured average zone of inhibition is gathered in Table 1. Based on the results presented below, it can be seen that moringa oil and inca inchi oil have no significant effect on the growth of the analyzed bacterial strains. On the other hand, tamanu oil inhibits the growth of the *S. epidermidis* bacterial strain.

### 3.2. Creaming Index

The creaming index is a measure of stability estimated by visual observation. Photographs of the emulsions were taken at the following intervals: immediately after preparation, after 24 h, after one week, after 2 weeks, and after 4 weeks (Figures S4 and S5), and the calculated creaming indexes are shown in Table 2 and Table 3.

In the case of the lecithin-stabilized emulsions, the samples with a 2.5% concentration of inca inchi oil and tamanu oil had the highest stability, as assessed by visual observation. Additionally, it was observed that the higher the concentration of oil within the sample, the lower the creaming index, indicating a loss of stability in the system. It should be noted that the amount of the emulsifier (lecithin) was constant, regardless of the amount of oil added, and it was 0.1 mL.

For emulsions with added casein, the best stability of the system was observed for the samples with 2.5% inca inchi oil and moringa oil as well. The amount of casein added was also constant for each sample. When comparing the emulsions based on visual observation, in terms of the added emulsifier, better CI results were noted for samples stabilized with lecithin than for samples stabilized with casein.

### 3.3. Droplets Size Distribution

Three measuring repetitions of the particle size distribution within the emulsion samples were performed. The collected results are presented graphically in Figure 1 and Figures S6–S8. From the graphs, it can be seen that the largest number of particles is between 100 and 1000 nm.

In the cases of the emulsions with the lowest oil concentration, i.e., 2.5% (*v*/*v*), the particle size discrepancy is the smallest, which indicates a better homogenization of the system. For samples that contain a 2.5% oil phase (*v*/*v*), it can be seen that the particle size distribution profile shows greater symmetry for emulsions with lecithin added as a stabilizer than for casein-stabilized ones. That behavior is particularly noticeable for samples loaded with moringa oil. For the emulsions with a 5% (*v*/*v*) oil phase, an increase in particle size was noticed. For samples stabilized with lecithin, the particle size distribution is more favorable due to a shift toward a smaller particle size that is shown in the collected graphs. The largest particle size was observed for samples with the highest oil phase concentration, i.e., 10% (*v*/*v*). This is undesirable for cosmetic and pharmaceutical applications, as an increase in particle size negatively affects the absorbability of the emulsion.

The predominant particle size was determined to illustrate which particles are most abundant within a given sample. The smaller the diameter of oil particles dispersed in the solution, the more favorable the stability of a given emulsion. Thus, the emulsions with the lowest oil phase concentration are the best. The smallest dominant particle diameter (220.2 nm) was observed for all samples with oils in concentrations of 2.5% (*v*/*v*) with lecithin added, as well as for samples with inca inchi oil and tamanu oil (2.5% *v*/*v*) stabilized by casein. The highest dominant particle diameter (825.0 nm) was observed only for samples stabilized by casein (with 5% and 10% oil concentrations (*v*/*v*)). Moreover, when comparing the effect of the used emulsifier, lecithin-stabilized systems have a more favorable particle size distribution.

### 3.4. Zeta Potential Analysis

**Figure 2:**
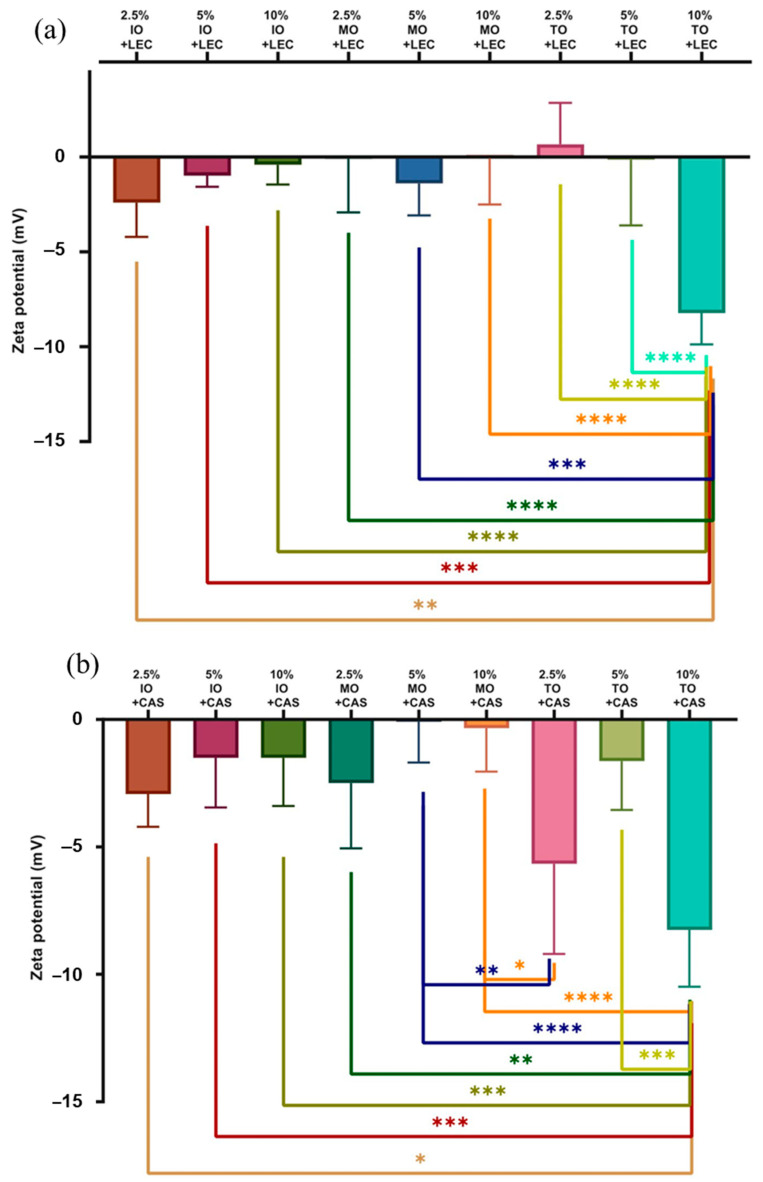
The zeta potential for all tested emulsions stabilized with (**a**) lecithin (LEC) or (**b**) casein (CAS); IO—inca inchi oil, MO—moringa oil, TO—tamanu oil; samples marked with different number of asterisks denote files of statistically significant differences.

The measurement of the zeta potential was repeated five times, and the obtained average values are shown in the graphs below (Figure 2). The higher the value of the potential (considered in absolute value), the more stable the system. As the absolute value of the zeta potential increases, the particles show better dispersion properties and an increase in electrostatic repulsion forces. The highest absolute value of the zeta potential was determined for the emulsion with a 10% tamanu oil phase, comparing both systems with lecithin (ζ = −8.232 mV) and with casein (ζ = −8.258 mV). If the zeta potential is close to zero, there is a tendency for particles to form aggregates and a deterioration of the emulsion stability to occur. From the tested samples, the result closest to zero was obtained for emulsions: with a 10% concentration of moringa oil and lecithin (ζ = −0.068 mV), with a 2.5% concentration of moringa oil and lecithin (ζ = −0.102 mV), with a 5% concentration of tamanu oil and lecithin (ζ = −0.138 mV), as well as with a 5% concentration of moringa oil and casein (ζ = −0.089 mV).

For samples with a concentration of oil phase equal to 2.5% (*v*/*v*), the highest absolute zeta potential value was observed for emulsions with inca inchi oil and casein, as well as with lecithin, and also for emulsions with moringa oil and casein. Samples with a concentration of oil phase equal to 5% (*v*/*v*) showed the lowest absolute value of zeta potential, except for emulsions with moringa oil and lecithin (where the |ζ| value was the highest) and emulsions with inca inchi oil and lecithin.

Emulsions with 10% of the tamanu oil phase showed the highest absolute value of zeta potential among all samples tested. In terms of the zeta potential analysis, the casein-stabilized emulsions showed more favorable results than the lecithin-stabilized samples.

### 3.5. Rheological Properties

**Figure 3:**
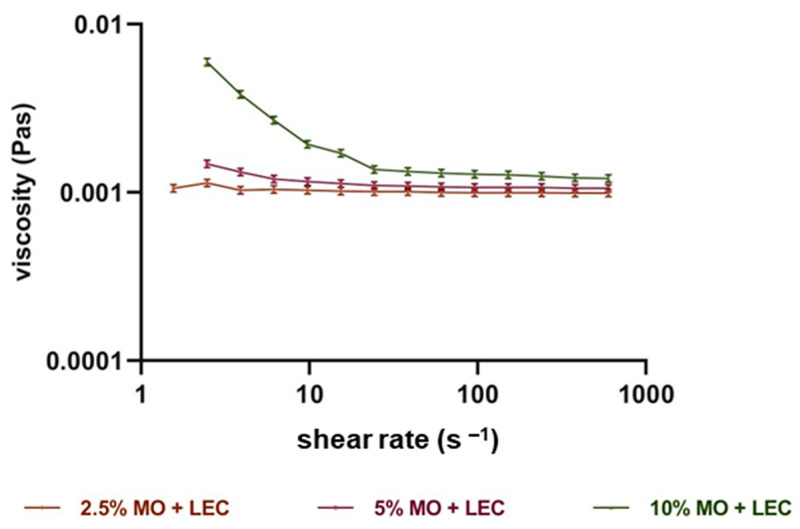
Viscosity curve for moringa oil (MO) emulsions with lecithin (LEC) as a stabilizer.

Dynamic viscosity and shear rate were determined, from which flow curves were drawn. The graphs are shown in Figure 3 and Figures S9–S14. When observing the curves of the relationship between the viscosity and the shear rate, it should be noted that they were not affected by the emulsifiers used, but the influence of the oil used was clearly marked. Taman oil, regardless of its concentration, showed the characteristics of a Newtonian fluid with constant viscosity values. Inca-inchi oil has some characteristics of a non-Newtonian fluid, which can be observed in its 10% oil phase content. Emulsions with 10% moringa oil content had particularly pronounced non-Newtonian characteristics. Both inca inchi oil and moringa oil emulsions exhibited shear-thinning fluid characteristics at large oil contents.

### 3.6. Metabolic Activity of Individual Strains

**Figure 4:**
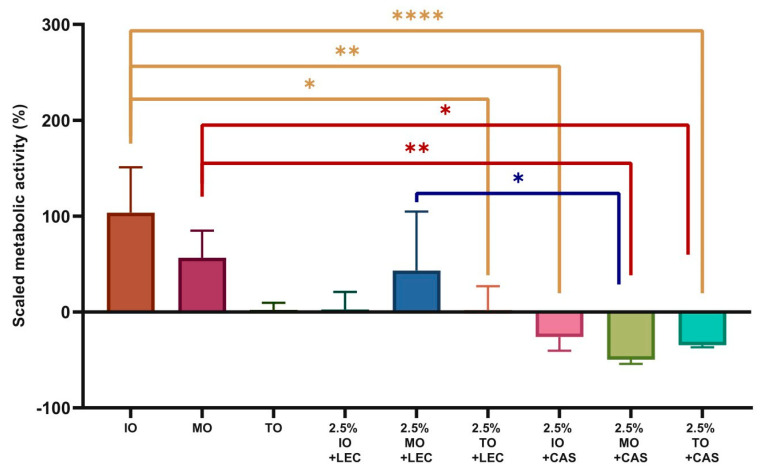
Relative metabolic activity for the *C. albicans* with IO—inca inchi oil, MO—moringa oil, TO—tamanu oil; lecithin—LEC; casein—CAS; samples marked with different number of asterisks denote files of statistically significant differences.

The metabolic activity of the strains was then calculated based on the results obtained, relating the results to a control sample containing a culture medium and the given strain of microorganisms. The calculated, scaled metabolic activity is shown in the following graphs (Figure 4 and Figures S15–S20). Negative values on the graphs indicate microbial cell death. Such an effect can be observed only in the cases of emulsions with a 2.5% oil phase (of any of the chosen oils), as shown in Figure 4. From the presented graphs, it can be seen that the emulsion with a 2.5% moringa oil concentration stabilized with casein is the most versatile because it shows antimicrobial properties against both Gram-negative bacteria (except *Pseudomonas aeruginosa*) and Gram-positive bacteria, as well as selected fungus cells. As can be seen, the effect of the individual oils varies against the strains tested, and only against some did they show an antibacterial effect. Moringa oil had the strongest effect against *E. coli*, reducing its metabolic activity by almost 50%. The presence of the emulsifier had a negative effect on cell growth, which may be explained by the increased biodegradability of the hydrophobic oil components towards the cells. However, emulsions with casein showed greater toxicity to *C. albicans* or *S. epidermidis*. The relatively high resistance of the *P. aeruginosa* strain appears to be specific to this particular strain. Perhaps its increased resistance is due to the ability of many strains of this species to produce their own biosurfactants and biodegrade oils (including mineral oils), which may imply that this species is better adapted to growth in the presence of hydrophobic compounds and surfactants/emulsifiers.

## 4. Discussion

In the first stage of this work, the antibacterial properties of the chosen three oils were verified. Based on the aforementioned presented results in Table 1, one can see that moringa oil and inca inchi oil have no significant effect on the growth of the analyzed bacterial strains. Tamanu oil, on the other hand, inhibits the growth of Gram-positive bacteria strains, like *S. epidermidis*, *S. aureus*, as well as *B. cereus*. The antimicrobial effect of tamanu oil on these and other bacterial species is also confirmed by the research of Léguillier et al. [22], although they found that the bactericidal effect strongly depends on the place of origin of the oil. The literature study by Urbankova et al. [15] indicates the antimicrobial properties of emulsions with tamanu oil and casein against Gram-positive bacterial strains (*S. aureus* and *B. cereus*), which, however, could not be observed during the course of the study in the present work. This indicates that compounds present in oils in relatively small amounts, and whose content strongly depends on where the raw material is harvested are responsible for antimicrobial properties [22]. This may also explain why the studies described earlier did not show an antibacterial effect of moringa oil (except in the case of *E. coli*), although Elgamily et al. [23] observed it against *S. aureus* or *Streptococcus mutans* (though not against *C. albicans*). Also worth mentioning is van den Berg and Kuipers’ [24] study on *Moringa oleifera* extracts, which shows that not only the raw material but also the method of extraction determines the antibacterial effect.

The emulsions’ stability results obtained were inconclusive, as the analysis of particle size distribution and creaming index indicated more favorable parameters for low-concentration systems stabilized with lecithin, while the analysis of zeta potential showed the highest stability for emulsions of 10% with the addition of casein. The creaming index increased as the amount of added oil increased (see Table 2). The destabilization of the emulsion was a direct result of the oil content since the amount of the emulsifier added was constant. Referring to the studies conducted for emulsions with tamanu oil and casein, it can be concluded that the use of a larger amount of stabilizing agent with an increase in oil concentration has a beneficial effect on the obtained creaming index value [15]. Due to the emulsifier used, lecithin showed better stabilizing properties. Lecithin, consisting mainly of phospholipids, contains both a lipophilic part, in the form of fatty acid groups, and a hydrophilic part, in the form of phosphorus-based esters, thanks to its unique structure. Casein, on the other hand, is a milk protein that also exhibits good emulsifying properties due to the existence of hydrophilic and lipophilic groups. Studies also indicate the benefits of combining casein with lecithin to form protein/phospholipid complexes that improve the stability of o/w systems [25].

The conducted tests made it possible to evaluate the emulsifying properties of the various oils. The samples with inca inchi oil were emulsified particularly well, which was indicated in visual observation and documented in the photographs. Much more significant destabilization was observed in samples using moringa oil and tamanu oil. The main reason for the observed different susceptibility to emulsification may be the different composition of these oils, primarily the ratio of saturated and unsaturated fatty acids and also shorter and longer fatty acids [26]. Additionally, studies on emulsions with tamanu oil indicate its poor emulsifying properties. It has been shown that oils with high viscosity tend to form emulsions with a larger particle size [15]. The most viscous of the oils applied is tamanu oil, which is roughly four times more viscous than inca inchi oil [3,27,28]. It is worth mentioning that increasing the power and time of sample processing above a certain threshold negatively affects the emulsion stabilization properties [29]. The rheological properties of sacha inchi oil emulsions were studied by González-Cardozo et al. [16], who found that the emulsions are shear-thinning fluids. However, the emulsifiers used were maltodextrin and a modified starch, which may have had an influence on the slope and shape of the curve. It is worth mentioning that when studying the properties of moringa oil emulsions (stabilized by the addition of stearic acid, stearyl alcohol, cetyl alcohol, and sorbitan ester 80), Athikomkulchai et al. [30] found that they are shear-thinning systems, as well.

## 5. Conclusions

The presented study delves into the analysis of various emulsions composed of natural oils to ascertain their potential antimicrobial properties. The investigation unveiled a correlation between the oil concentration and stability, as assessed by the creaming index of the samples. In lecithin-stabilized emulsions, the sample featuring a 2.5% concentration of inca inchi oil and tamanu oil exhibited the highest stability. Meanwhile, among casein-stabilized emulsions, superior stability was observed in samples with a 2.5% concentration of inca inchi oil and moringa oil. These findings underscored the intricate interplay between oil concentration and the choice of stabilizer in shaping emulsion stability. Moreover, the potential antimicrobial properties of emulsions formulated with natural oils were observed. The amassed data from this study represent a valuable foundation for future research endeavors and offer insights into the development of natural oil-based emulsions with antimicrobial properties. The conducted studies provide valuable results, allowing us to identify the most favorable compositions of emulsions containing moringa, tamanu, and inca inchi oils. Due to their nutritional value and antibacterial properties, they can become a useful component in dietary supplements, food additives, pet food, as well as cosmetics. Such emulsions can become a useful vector for getting unsaturated fatty acids, vitamins, etc., into an organism.

## Figures and Tables

**Table 1:** Collected measurements of inhibition zones of bacterial growth under the influence of tested cosmetic oils. Where ‘-’ signifies the absence of any discernible zone of growth inhibition, ‘+’ denotes growth inhibition in the zone below 5 mm, ‘++’ in the zone between 5 mm and 10 mm, ‘+++’ in the zone between 10 mm and 20 mm.

Strain	Tamanu Oil	Moringa Oil	Inca Inchi Oil
*P. aeuruginosa*	+	-	+
*P. fluorescens*	-	-	+
*E. coli*	+	+	+
*S. epidermidis*	+++	+	+
*S. aureus*	+++	-	+
*B. cereus*	+++	++	-
*C. albicans*	+	+	+

**Table 2:** The creaming index (CI) after 1, 7, 14, and 28 days calculated for emulsions with lecithin added.

Vegetable Oil with the Addition of Lecithin	Emulsion Concentration	Creaming Index [%]			
		CI_1_	CI_7_	CI_14_	CI_28_
**Inca inchi**	2.5%	100	100	100	100
	5%	100	100	97.5	95
	10%	100	97.5	95	95
**Moringa**	2.5%	100	97.5	95	95
	5%	100	97.5	95	90
	10%	100	97.5	95	90
**Tamanu**	2.5%	100	100	100	100
	5%	100	100	97.5	95
	10%	97.5	97.5	95	90

**Table 3:** The creaming index (CI) after 1, 7, 14, and 28 days calculated for emulsions with casein added.

Vegetable Oil with the Addition of Lecithin	Emulsion Concentration	Creaming Index [%]			
		CI_1_	CI_7_	CI_14_	CI_28_
**Inca inchi**	2.5%	100	97.5	97.5	97.5
	5%	100	97.5	95	95
	10%	95	90	90	90
**Moringa**	2.5%	100	97.5	97.5	97.5
	5%	95	95	95	95
	10%	95	90	90	90
**Tamanu**	2.5%	100	97.5	97.5	95
	5%	95	95	95	90
	10%	90	90	90	90

## Data Availability

Data is contained within the article.

## Supplementary Materials

The following supporting information can be downloaded at: https://www.mdpi.com/article/10.3390/foods13010062/s1, Figures S1–S20.
