# Supplementary material for: The Bioactivity and Physicochemical Properties of Emulsions Based on Tamanu, Moringa, and Inca Inchi Oils
Source: Foods. 2023 Dec 22;13(1):62. doi: 10.3390/foods13010062 (PMC10778635; doi:10.3390/foods13010062)
Supplement: Supplementary file 1 [file foods-13-00062-s001.zip › foods-2737344-supplementary.pdf]

Supplementary Materials

1. Gas Chromatography analysis

Analysis of the composition of selected vegetable oils was performed using the gas chromatography technique. A Pegasus 4D GCxGC-TOMF/MS gas chromatograph from LECO (USA) was used to assess the oils compositions.

A 60 m × 0.25 mm × 0.25 μm VF-5MS fused silica chromatography column was applied for the analysis. It is a 5% phenyl/95% dimethylpolysiloxane stationary phase column. The sample injection volume was 0.5 μl, and the flow rate of helium, used as a carrier gas, was 1 ml/min. The injection temperature was 240°C, and the ion source temperature was 200°C. The oven temperature was initially set at 70°C, maintaining isothermal conditions for 3 min, with an increase of 10°C/min to 240°C, ending at 280°C maintaining a constant temperature for 9 min.

Samples were derivatized before chromatographic analysis to obtain trimethylsilane derivatives of the analyzed compounds, showing lower polarity and boiling point. N,O-bis-trimethylsilyltrifluoroacetamide (BSTFA) was used as a silylating reagent. Therefore a dose of 200 μl of BSTFA was added per 50 mg of each oil sample. The samples were then incubated for one hour at 65°C before placing into a tray of the gas chromatograph.

Chromatographic analysis of inca inchi oil (Figure S1) showed the presence of the following fatty acids: pentanoic acid (valerian), octadecanoic acid (stearic acid), alpha-linolenic acid, alpha-linolenic acid and hexadecanoic (palmitic) acid.

The analysis of moringa oil (Figure S2) revealed the presence of the following fatty acids: hexadecanoic (palmitic) acid, octadecanoic (stearic) acid, eicosanoic (arachidic) acid, docosanoic (behenic) acid, and oleopalmitic acid.

Moreover, the results obtained for tamanu oil (Figure S3) showed the presence of the following fatty acids: butanoic acid, heptadecanoic acid (margaric), hexadecanoic acid (palmitic), eicosanoic acid (arachidic), and alpha-linolenic acid.

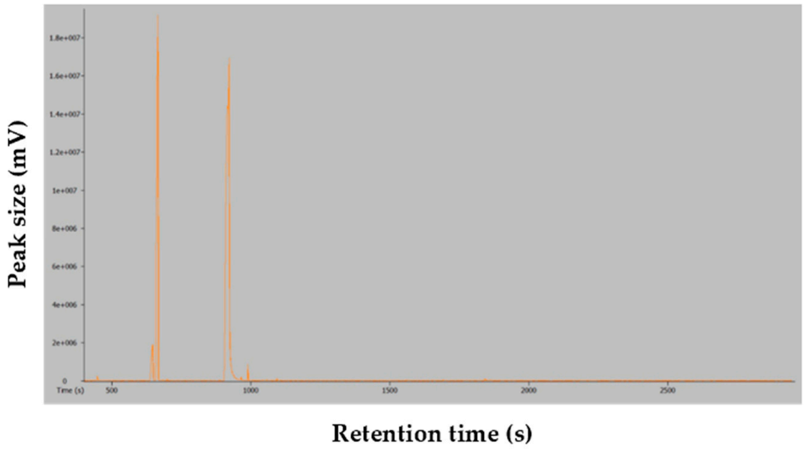

Figure S1 Chromatogram of a sample of inca inchi oil.

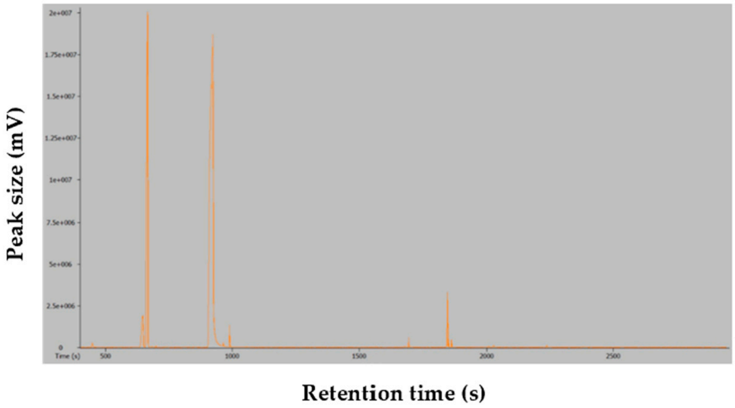

Figure S2 Chromatogram of a sample of moringa oil.

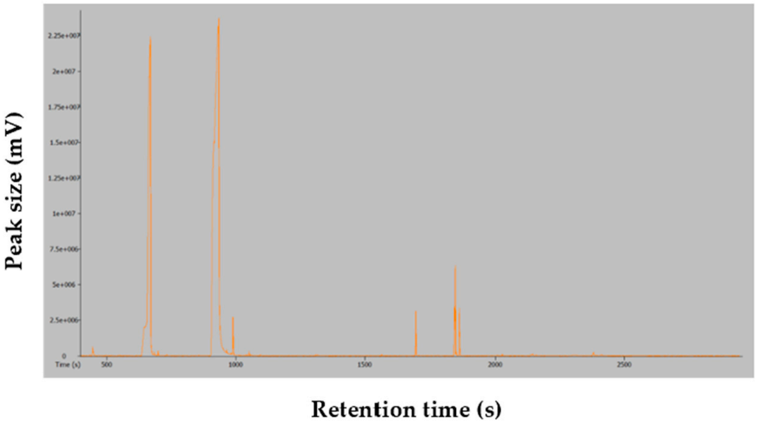

Figure 1 Chromatogram of a sample of tamanu oil.

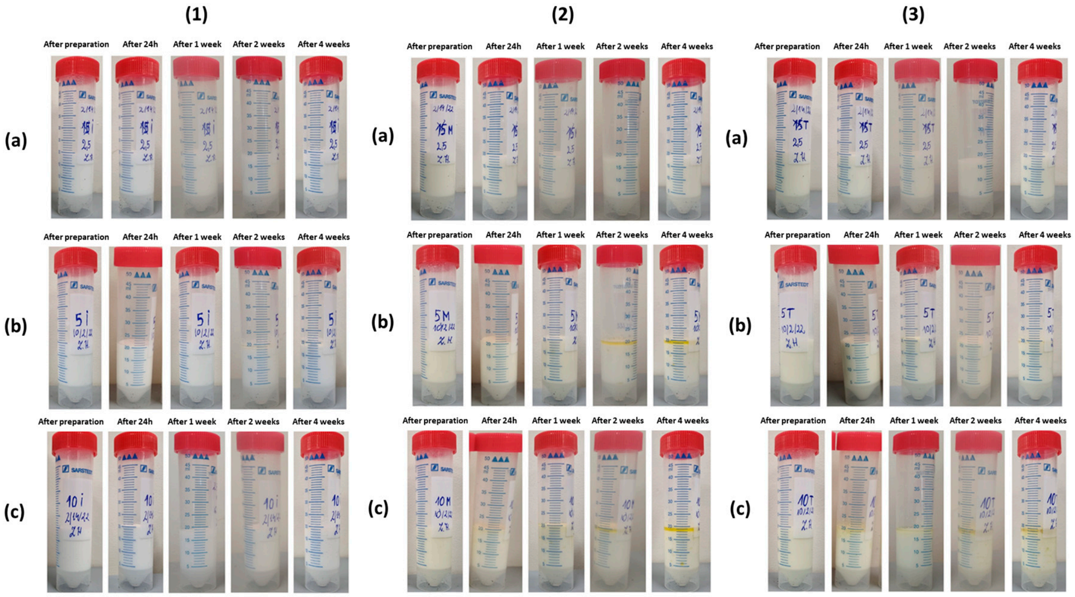

Figure S4 Photographs of samples taken at given time intervals for emulsions with (1) inca inchi, (2) moringa, and (3) tamanu oil, with the addition of lecithin, at concentrations of (a) 2.5%, (b) 5% and (c) 10% (v/v) of an oil phase.

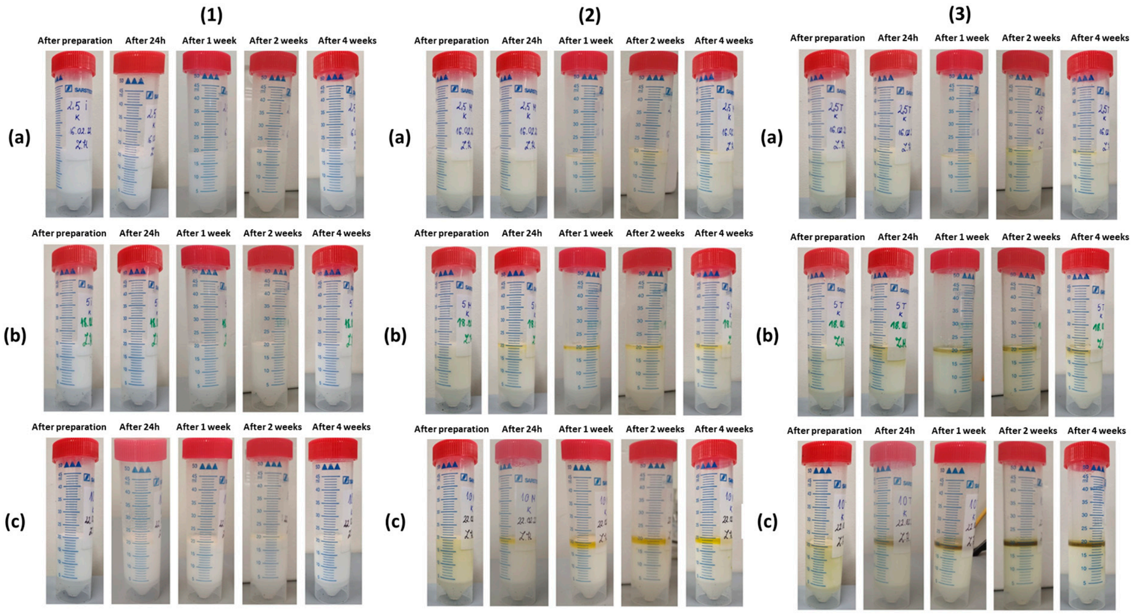

Figure S5 Photographs of samples taken at given time intervals for emulsions with (1) inca inchi, (2) moringa, and (3) tamanu oil, with the addition of casein, at concentrations of (a) 2.5%, (b) 5% and (c) 10% (v/v) of an oil phase.

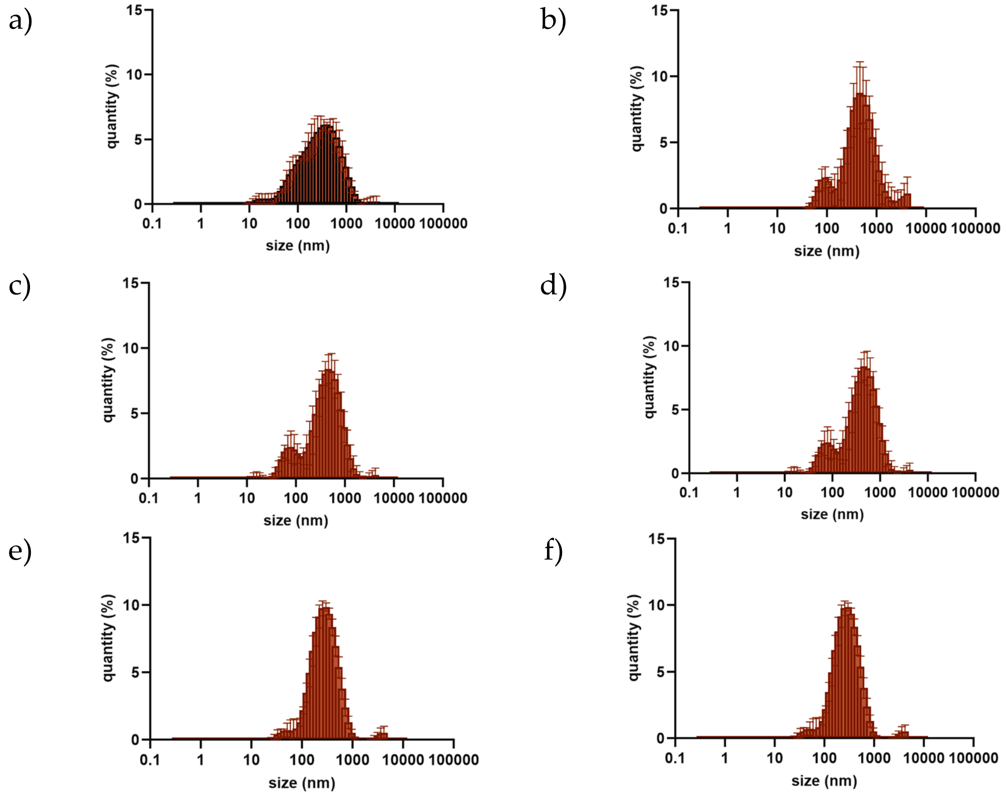

Figure S6 Particle size distribution for o/w emulsions with 10% oil emulsions with inca inchi oil (a, b); moringa oil (c, d); tamanu oil (e, f) stabilized with lecithin (a, c, e) or casein (b, d, f)

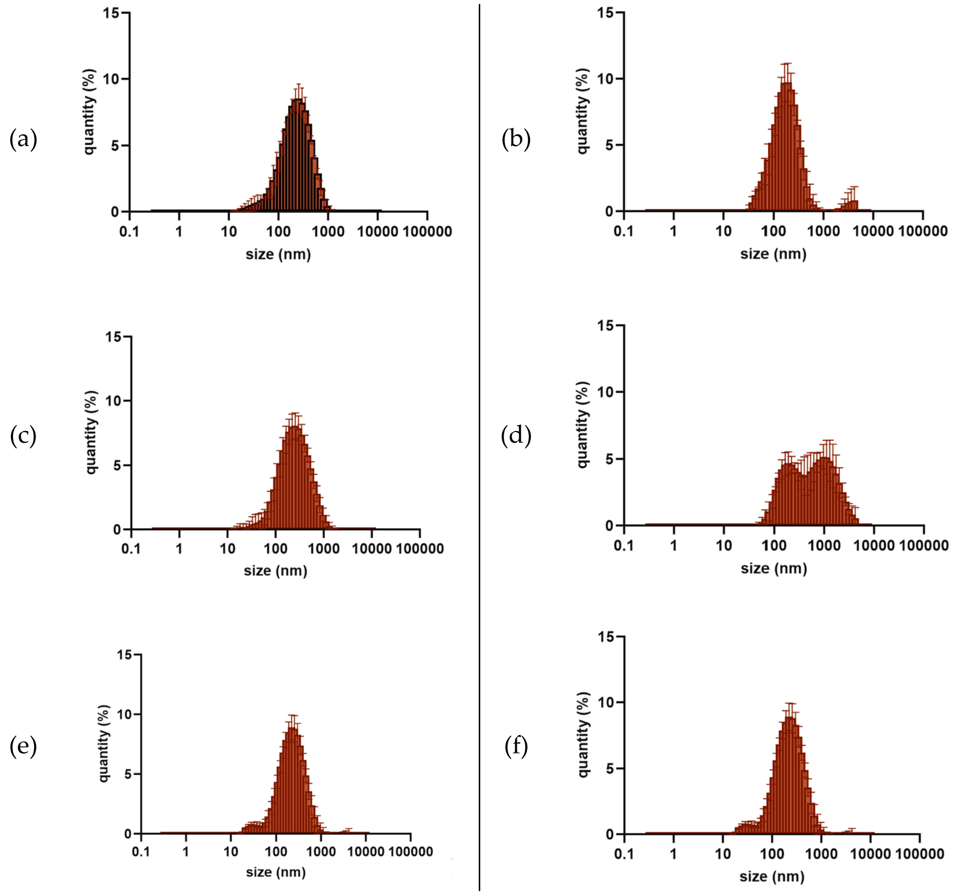

Figure S7 Particle size distribution for o/w emulsions with 2.5% oil emulsions with inca inchi oil (a, b); moringa oil (c, d); tamanu oil (e, f) stabilized with lecithin (a, c, e) or casein (b, d, f)

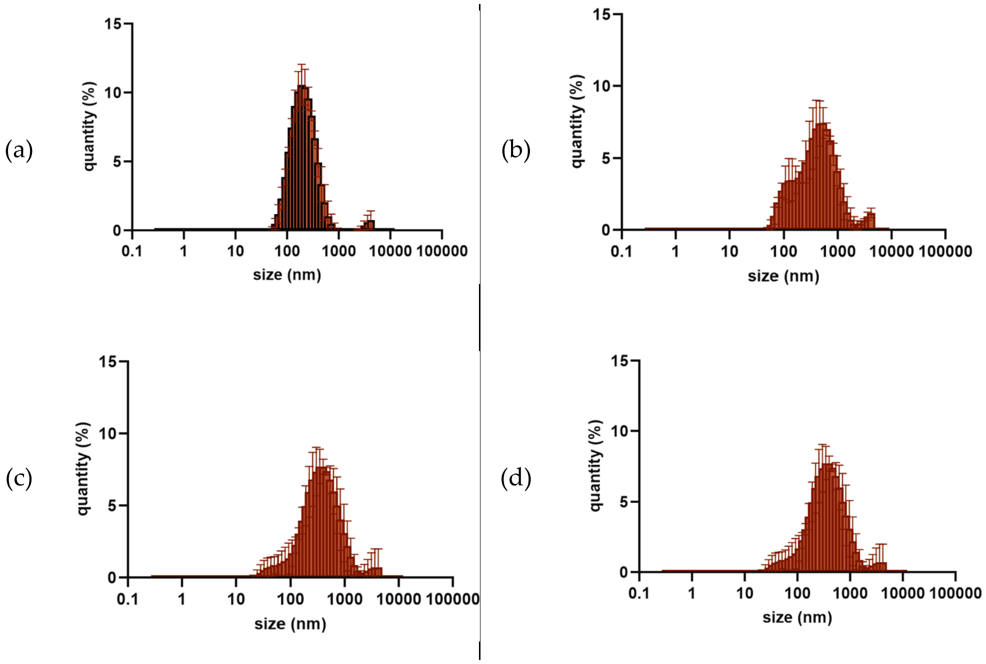

(e)

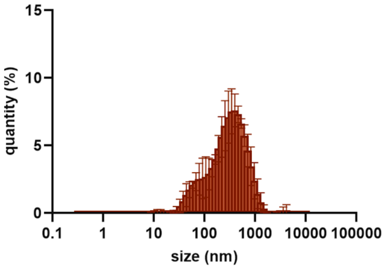

(f)

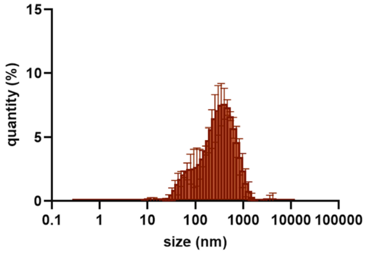

Figure S8 Particle size distribution for o/w emulsions with 5% oil emulsions with inca inchi oil (a, b); moringa oil (c, d); tamanu oil (e, f) stabilized with lecithin (a, c, e) or casein (b, d, f)

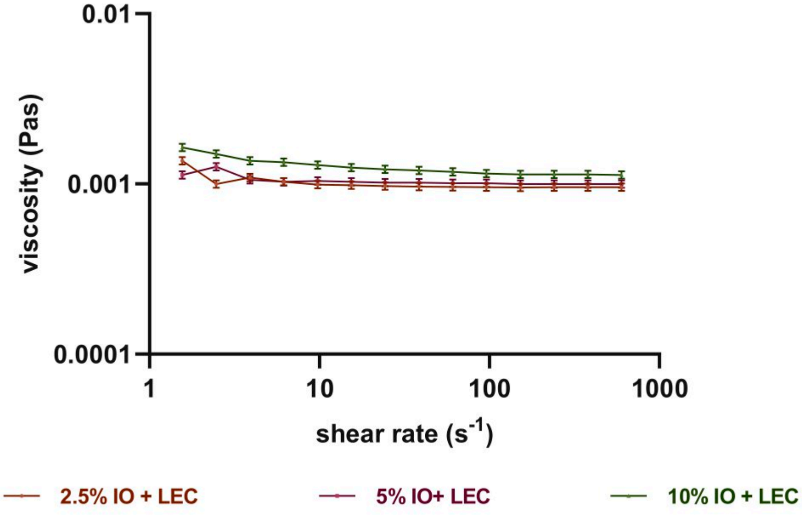

Figure S9 Viscosity curves for emulsions with inca inchi oil and lecithin. Where: 2.5%IO+LEC -2.5% inca inchi oil+lecithin, 5%IO+LEC -5% inca inchi oil+lecithin, 10%IO+LEC -10% inca inchi oil+lecithin.

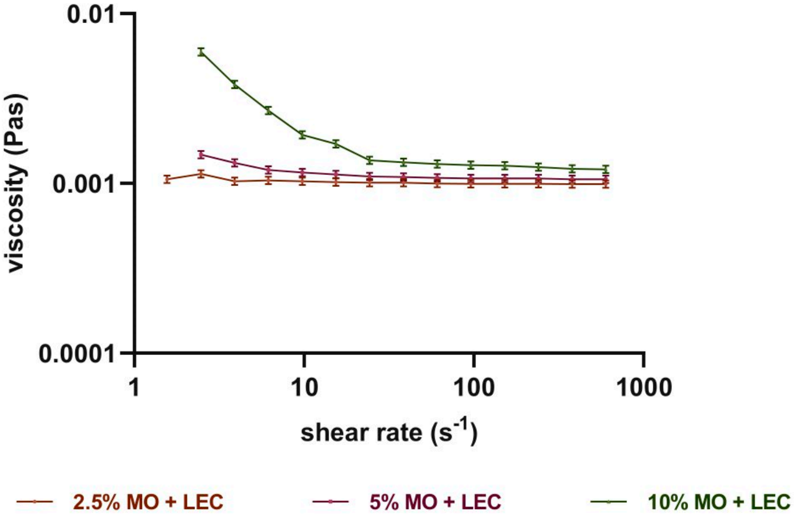

Figure S10 Viscosity curves for emulsions with moringa oil and lecithin. Where: 2.5%MO+LEC - 2.5% moringa oil+lecithin, 5%MO+LEC -5% moringa oil+lecithin, 10%MO+LEC -10% moringa oil+lecithin.

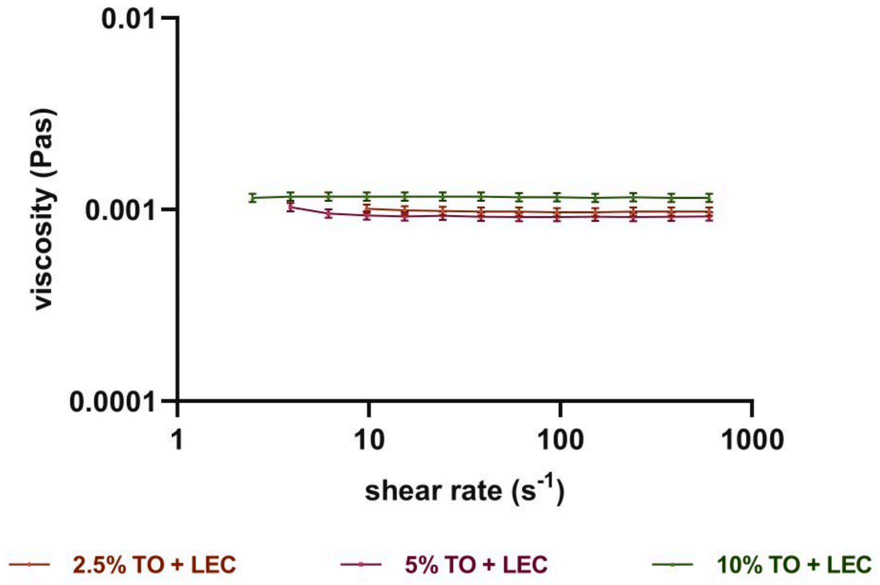

Figure S11 Viscosity curves for emulsions with tamanu oil and lecithin. Where: 2.5%TO+LEC -2.5% tamanu oil +lecithin, 5%TO+LEC -5% tamanu oil+lecithin, 10%TO+LEC -10% tamanu oil+lecithin.

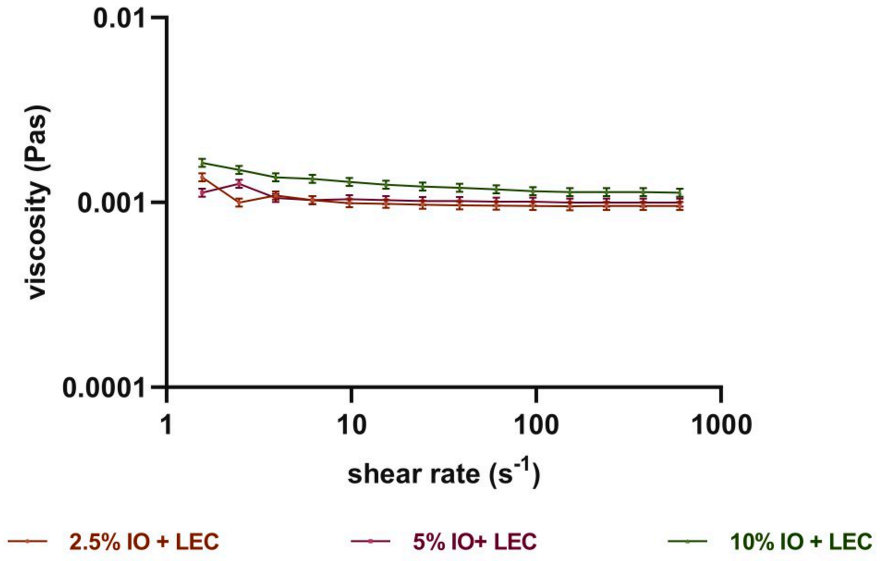

Figure S12 Viscosity curves for emulsions with moringa oil and casein. Where: 2.5%IO+CAS -2.5% inca inchi oil+casein, 5%IO+CAS -5% inca inchi oil+casein, 10%IO+CAS -10% inca inchi oil+casein.

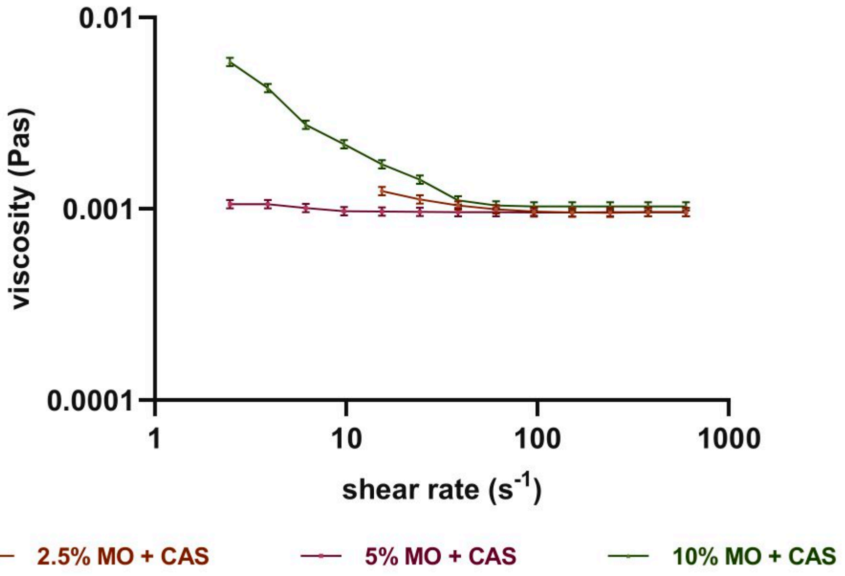

Figure S13 Viscosity curves for emulsions with moringa oil and casein. Where: 2.5%MO+CAS -2.5% moringa oil+casein, 5%MO+CAS-5% moringa oil+casein, 10%MO+CAS -10% moringa oil+casein.

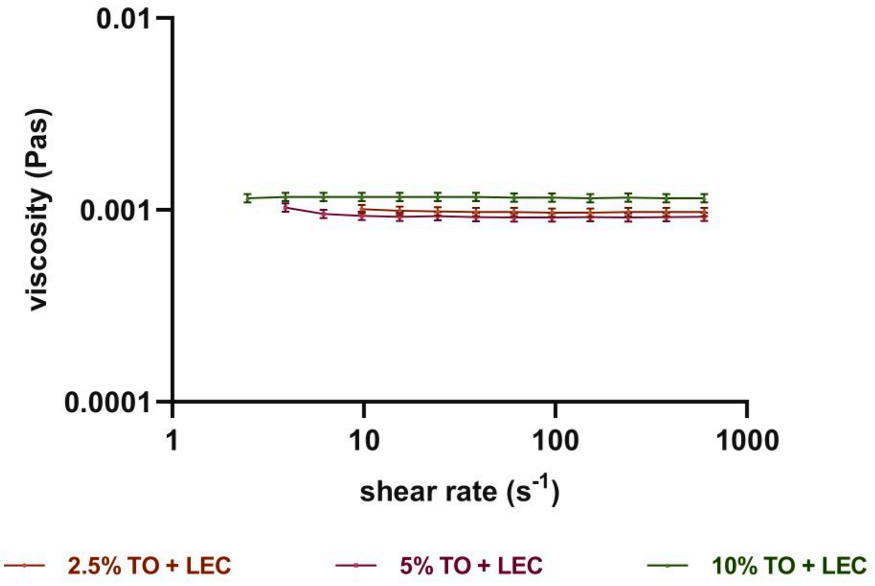

Figure S14 Viscosity curves for emulsions with tamanu oil and casein. Where: 2.5%TO+CAS-2.5% tamanu oil +casein, 5%TO+CAS -5% tamanu oil+casein, 10%TO+CAS-10% tamanu oil+casein.

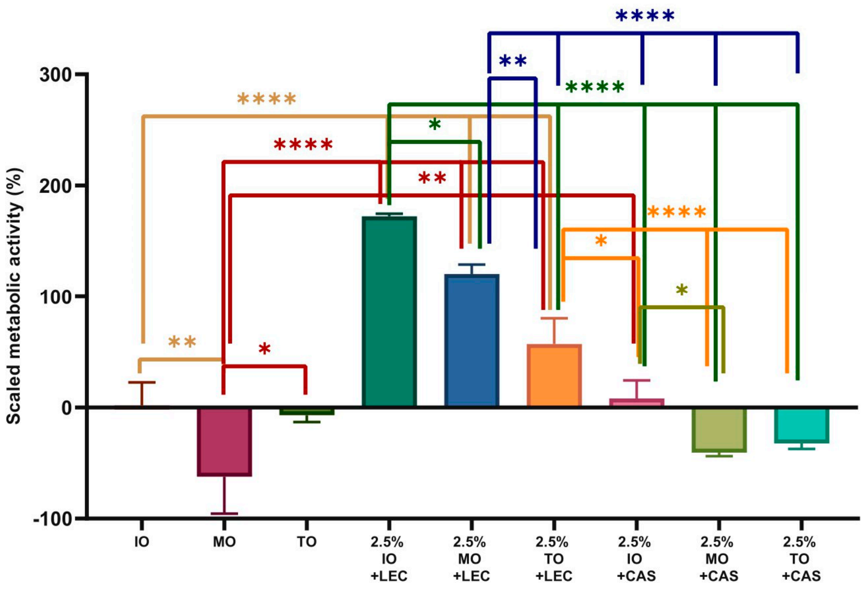

Figure S15 Relative metabolic activity for the *Escherichia coli* strain; IO – inca inchi oil, MO – moringa oil, TO – tamanu oil; lecithin – LEC; casein – CAS.

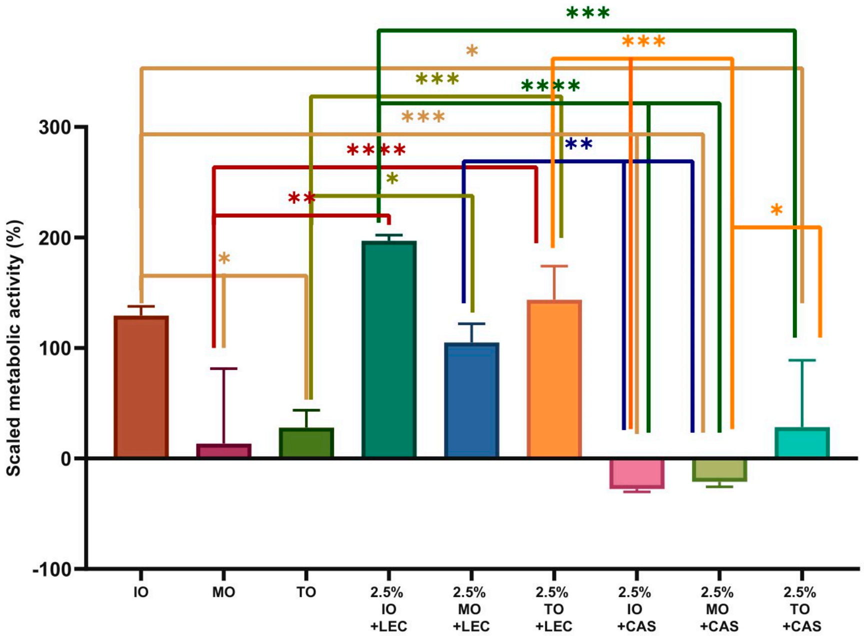

Figure S16 Relative metabolic activity for the *Pseudomonas fluorescens* strain; IO – inca inchi oil, MO – moringa oil, TO – tamanu oil; lecithin – LEC; casein – CAS.

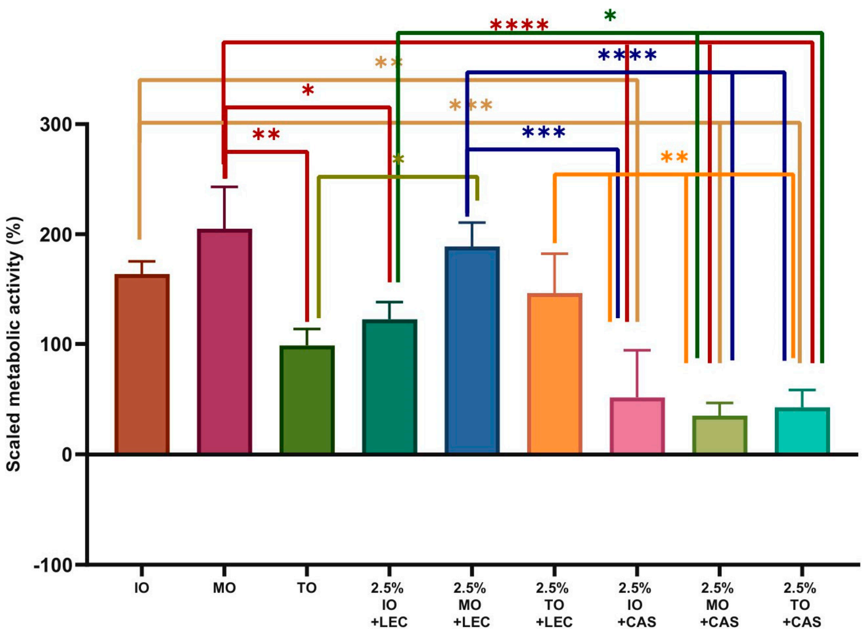

Figure S17 Relative metabolic activity for the *Pseudomonas aeruginosa* strain; IO – inca inchi oil, MO – moringa oil, TO – tamanu oil; lecithin – LEC; casein – CAS.

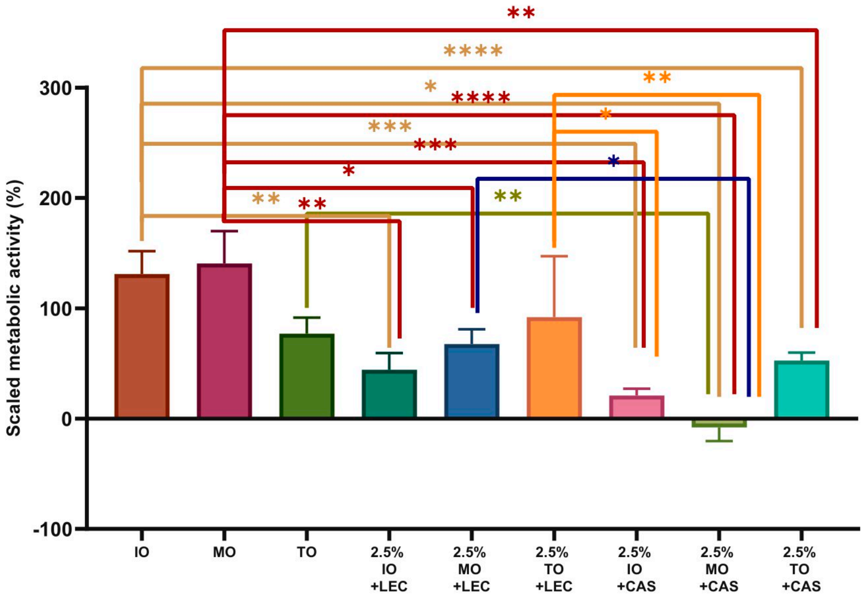

Figure S18 Relative metabolic activity for the *Bacillus cereus* strain; IO – inca inchi oil, MO – moringa oil, TO – tamanu oil; lecithin – LEC; casein – CAS.

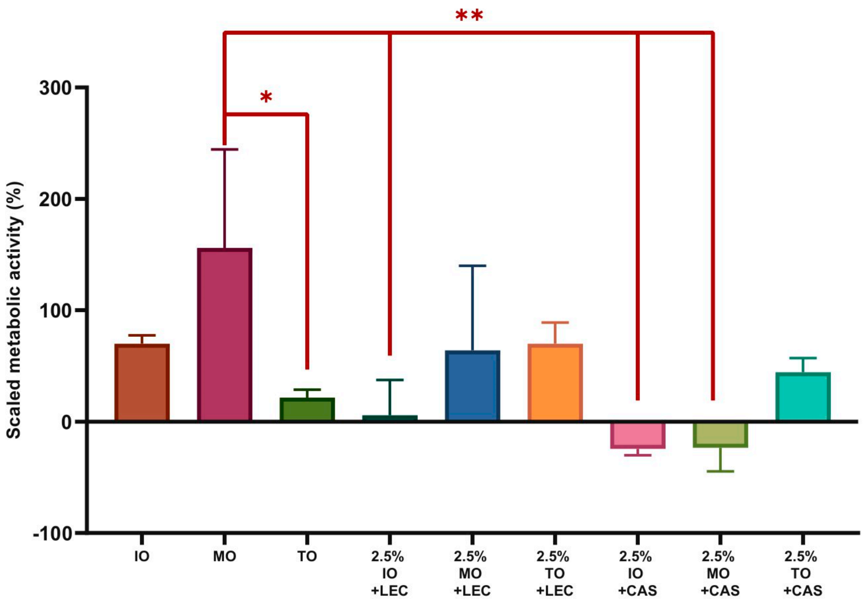

Figure S19 Relative metabolic activity for the *Staphyloccocus aureus* strain; IO – inca inchi oil, MO – moringa oil, TO – tamanu oil; lecithin – LEC; casein – CAS.

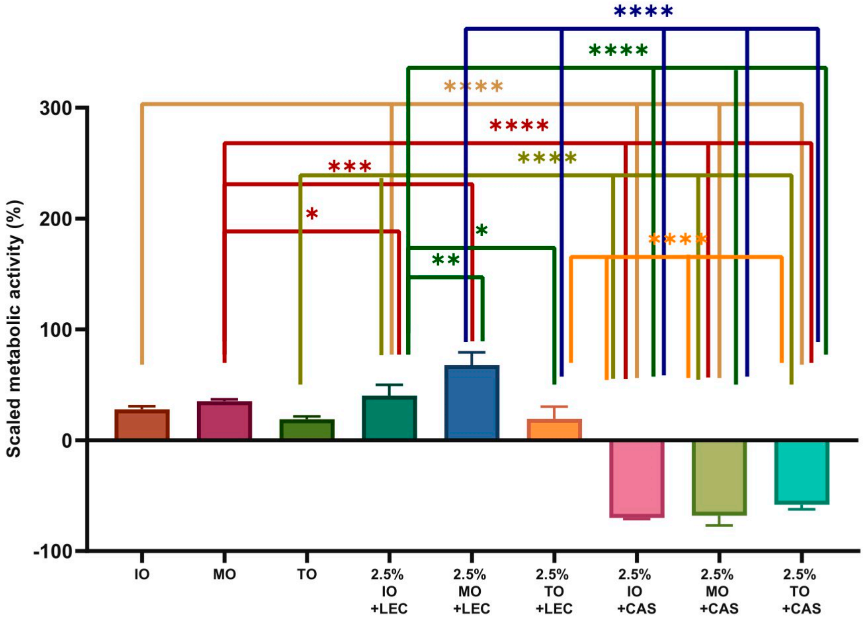

Figure S20 Relative metabolic activity for the *Staphylococcus epidermidis* strain; IO – inca inchi oil, MO – moringa oil, TO – tamanu oil; lecithin – LEC; casein – CAS.
